# Development and Application of a ZigBee-Based Building Energy Monitoring and Control System

**DOI:** 10.1155/2014/528410

**Published:** 2014-08-28

**Authors:** Changhai Peng, Kun Qian

**Affiliations:** ^1^School of Architecture, Southeast University, Nanjing 210096, China; ^2^Key Laboratory of Urban and Architectural Heritage Conservation (Southeast University), Ministry of Education, Nanjing 210096, China; ^3^IIUSE, Southeast University, Nanjing 210096, China; ^4^School of Automation, Southeast University, Nanjing 210096, China

## Abstract

Increasing in energy consumption, particularly with the ever-increasing growth and development of urban systems, has become a major concern in most countries. In this paper, the authors propose a cost-effective ZigBee-based building energy monitoring and control system (ZBEMCS), which is composed of a gateway, a base station, and sensors. Specifically, a new hardware platform for power sensor nodes is developed to perform both local/remote power parameter measurement and power on/off switching for electric appliances. The experimental results show that the ZBEMCS can easily monitor energy usage with a high level of accuracy. Two typical applications of ZBEMCS such as subentry metering and household metering of building energy are presented. The former includes lighting socket electricity, HVAC electricity, power electricity and special electricity. The latter includes household metering according to the campus's main function zone and each college or department. Therefore, this system can be used for energy consumption monitoring, long-term energy conservation planning, and the development of automated energy conservation for building applications.

## 1. Introduction

With the emergence of new and innovative technologies, living standards and quality of life have reached an all-time high. A significant part of the modern lifestyle is intertwined with the usage of electronic and electrical devices. However, increases in the utilization of electronics and electrical appliances have adversely resulted in an unprecedented increase in energy consumption. Subsequently, due to the demand-supply gap, the price paid by the end user continues to increase annually. As a result, there is a serious need to optimize energy consumption and develop more energy-efficient technologies and electronic systems. This need has resulted in the development of new fundamental and applied research fields in the area of energy conservation. Among these research areas, with the potential to result in significant developments in energy consumption, is the design of integrated advanced monitoring and control mechanisms with the capability to better monitor and control power consumption, so that users can easily measure the power consumption of electronic devices and optimize their usage to enhance their energy consumption performance [1].

With advancements in wireless technologies and through the implementation of distributed sensor networks, residential energy consumption systems are beginning to take advantage of these systems for reducing energy consumption and thus increasing energy efficiency. By eliminating the need to run wires in an existing facility, wireless technologies can help reduce the cost of construction in an “intelligent” building. Due to their small footprints, wireless nodes can be easily mounted without interruption of usage and without inconveniencing building occupants with renovations and changes. Another benefit of wireless technologies that makes them appropriate for residential use is their low energy consumption, as they can be powered by batteries with long service lives [2].

Table 1 outlines the key characteristics of some common wireless mechanisms [3]. From an application perspective, Bluetooth is intended for cordless mice, keyboards, and hands-free headsets. As an improved Bluetooth version, BLE (Bluetooth low energy) is intended to provide considerably reduced power consumption and cost while maintaining a similar communication range. The ultrawideband (UWB) is oriented to high-bandwidth multimedia links. The wireless universal serial bus (wireless USB) is the personal interconnect technology used to meet the needs of multimedia consumer electronics, PC peripherals, and mobile devices. Wi-Fi is directed at computer-to-computer connections as an extension or substitution for cabled networks [4, 5]. Infrared (IR) wireless approaches are used for short- and medium-range communications and control. Unlike radio-frequency (RF) wireless links, IR wireless links cannot penetrate walls or other obstructions [6].

In contrast to other listed wireless protocols in Table 1, ZigBee is designed for reliable wirelessly networked monitoring and control networks. An example of comparison between ZigBee and BLE, two most popular techniques for wireless measurement applications, demonstrates the reason why the former is favorable for our application. BLE is more oriented towards user mobility whereas ZigBee aims for automation and remote control. Bluetooth supports 8 nodes per network whereas ZigBee supports up to 255 nodes per network. In addition, the advantage of ZigBee in mesh networking capabilities allows itself to be very easy to install without the need for any special installation services. Therefore, ZigBee is more suitable for remote energy monitoring and control.

Due to the above reason, in this paper a ZigBee-based building energy monitoring and control system (ZBEMCS) is presented, which offers a promising solution for the aforementioned objective. For monitoring, the hardware is based on current and voltage measuring circuits, a microcontroller unit (MCU), a control module, and a ZigBee module. The current/voltage measuring circuit measures the current and voltage and sends the information to the MCU. The MCU checks for power abnormalities and sends information to the building server, where a database is maintained through ZigBee. For control, a relay is added to the power monitoring hardware. In the case of an emergency found by the MCU, the relay cuts the power supply to the electric building appliances after receiving the control command. A graphic user interface (GUI) software program is used as an interface between the user and the end devices. Subsequently, the user can control all electric appliances through a cell phone or a desktop or laptop computer.

## 2. Related Work

Energy monitoring is essential for understanding the sources of consumption inside a building and to take appropriate measures to save energy. Generally, building energy monitoring and control efforts can be divided in two broad categories: hardware- and software-based.

Hardware-based approaches focus on involving physical equipment such as smart plugs and smart plug strips for controlling information and communication technologies (ICTs) devices. The studies performed in [7–9] indicate the perspective of considerable savings. In addition, replacing equipment with more energy-efficient one can be effective, as observed in [10, 11], with savings around 40–60%. Kamilaris et al. [12] believed that the contribution of hardware-based methods for savings needs to be quantified. In this way, companies and organizations would be aware about the return of investment when considering any of these approaches.

Software-based techniques consider mainly power management (PM) and virtualization. Somniloquy [13] and SleepServer [14] are pioneering efforts regarding PM, claiming significant savings exceeding 60%. LiteGreen [15] and VMware [16] are dominating in the field of virtualization. Current commercial products for PM and virtualization are efficient and reliable, offering advanced features and large potential for savings.

A comparison among hardware and software-based techniques [11] shows that hardware-based approaches are more effective, for example, by replacing desktop computers with laptops. Other approaches stress the role of commercial buildings in smart grid scenarios [17, 18] and the importance of combining sensing with actuation [19].

Meanwhile, relevant efforts recognize the large impact of occupants, affecting 20–50% of total building's energy use [10, 20], and focus on motivating the occupants towards energy savings through suggestions and advice, timely and comparative eco-feedback techniques [21–23].

While hardware and software-based techniques can affect electricity consumption in a large degree, provisioning is crucial for conservation. Decisions made during the early design stage can influence about 60% of total energy usage life cycle, leaving the impact of user behavior and real-time control to the rest 40%. Still, even small savings can have significant effects on the overall costs of companies and on the environment [12].

Apparently, in order to achieve standardized, effective, and objective green standards for commercial buildings and miscellaneous electric loads, international energy policies and regulations need to be defined by stakeholders and key players, involving legislative measures, economic instruments, voluntary agreements, and technology and innovation specifications.

Lastly, embedded ICTs, although increasing their collective energy consumption globally, are expected to play a crucial role in energy efficiency across the economy, helping office equipment to operate in a more intelligent, automated, and efficient way.

Compared with the abovementioned related work, the contributions of our proposed system combine hardware and software-based techniques. Firstly, our system accommodates both traditional building energy meters and environmental sensors, for wireless data transmission and management in an integrated framework, which empowers the collection and monitoring of various types of measurements that reflect the energy consumption and environmental status of buildings. Secondly, the system is further extended with web-based management software, which offers rich analysis and advanced report functions for monitoring both energy consumption and environment.

## 3. System Architecture

**Figure 1:**
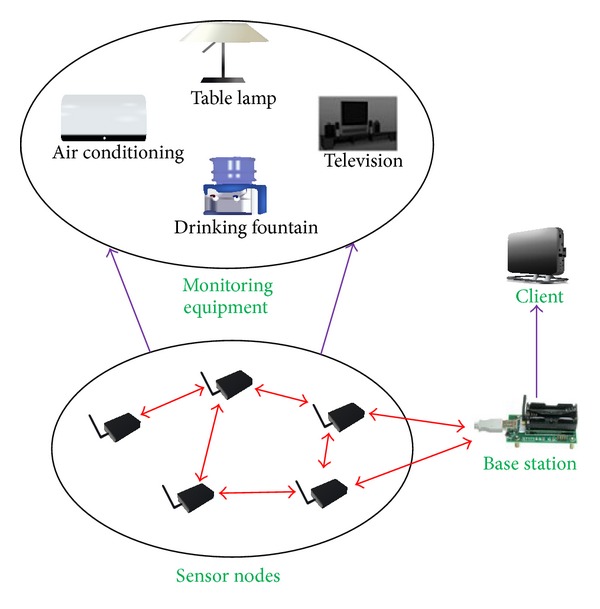
Architecture of the ZBEMCS.

The ZBEMCS consists of a gateway, a base station, and sensors, as shown in Figure 1. The gateway is also named the client, and its purpose is to connect sensor nodes to an existing Ethernet network. The base station provides a connection between the sensor nodes and the gateway. The sensors monitor and control the energy usage of the electrical equipment and transmit data to the base station.

### 3.1. Gateway

For this implementation, the gateway is assigned the name SQ120 (“Client” in Figure 1) and is based on an Intel IXP420 XScale processor running at 266 MHz, which features one wired Ethernet port and two USB 2.0 ports. The device is further equipped with 8 MB of the program FLASH, 32 MB of RAM, and a 2 GB USB 2.0 system disk. SQ120 runs the Debian Linux operating system, which is a full-fledged standard Linux distribution for the ARM architecture that comes preloaded with Crossbow's sensor network management and data visualization software packages, XServe and MoteExplorer. These programs are started automatically at the boot time of the SQ120. To set up a sensor network gateway configuration, a base station should be plugged into the secondary USB port of the SQ120. SQ120 contains a built-in web server (MoteExplorer) and a sensor network management tool (XServe). The latter can automatically identify what types of sensor boards are plugged into the nodes of the wireless sensor network and instructs MoteExplorer to display the data accordingly [24].

### 3.2. Base Station

The base station is the monitoring and controlling center of all branch circuits and the gateway for external communication and the user interface; its main functions are as follows [25]:

1. executing control instructions through the Internet;
2. monitoring the energy consumption of the sensor nodes;
3. calculating the remaining power capacity of each branch circuit;
4. indicating all energy consumption information.

**Figure 2:**
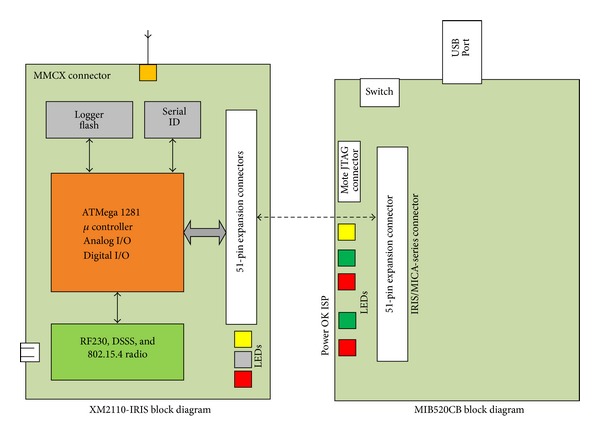
Structure of the base station.

As shown in Figure 2, the base station, which is a full function device (FFD), consists of the mote processor/radio platforms (XM2110) and a gateway (MIB520CB) via a 51-pin expansion connector. Thus, the base station is configured as a ZigBee coordinator (ZC) of WSNs. The base station receives the data sent by all nodes in the network and sends a message across the USB connection to the computer. The base station runs the Debian Linux operating system preloaded with Crossbow's sensor network management and data visualization software packages, including EcoView and Xserve [26].

**Figure 3:**
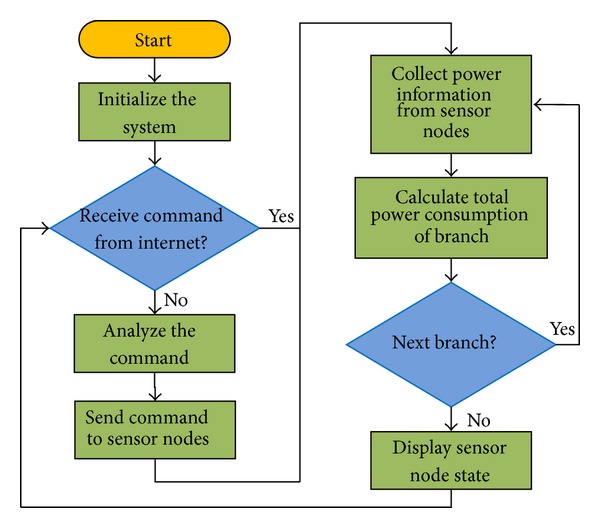
Software flow chart for the base station.

Figure 3 shows a software flow chart of the base station.

### 3.3. Sensor Nodes

**Figure 4:**
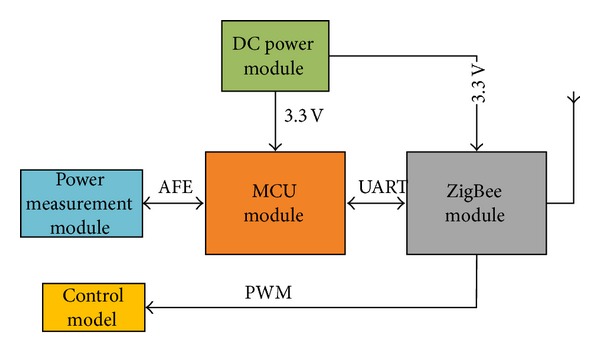
Block diagram of the sensor node.

The sensor node, which is the measure and control node, is shown in Figure 4. The sensor node is comprised of a direct current (DC) power module, a MCU, an alternating current (AC) power control module, and a ZigBee module. The MCU module communicates with the power measurement module by an analog front end (AFE) and with the ZigBee module through universal asynchronous receiver/transmitter (UART) interfaces. Communication between the ZigBee module and the control module is achieved by the pulse width modulation (PWM) technique. The main functions of the sensor node are as follows [25]:

1. measurement of power parameters, such as the voltage, current, and power of the outlet;
2. control of the power output of the outlet;
3. security protection from overload;
4. transmission of the information of each node to the base station through ZigBee.

#### 3.3.1. DC Power Module

**Figure 5:**
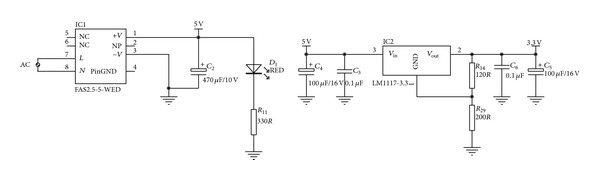
Circuit structure of the DC power module.

The main function of the DC power module is to convert 220 V of AC power into 5 V and 3.3 V of DC power to provide the operating power for all modules in the sensor node. The module's circuit structure is shown in Figure 5. The AC 220 V is converted by switching the power module to DC 5 V and 3.3 V by a linear regulator.

#### 3.3.2. Power Measurement Module

**Figure 6:**
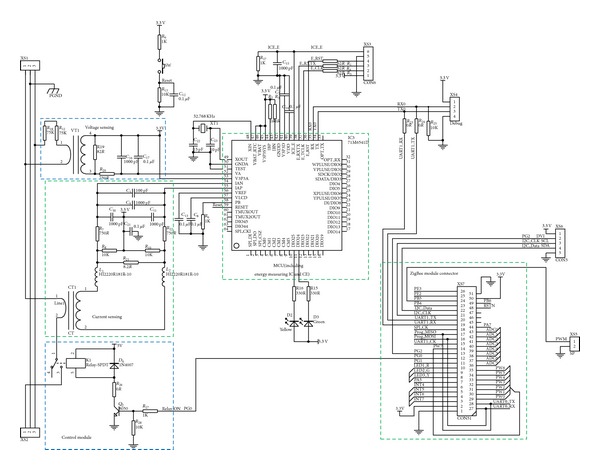
Circuit diagram of the sensor node.

The power-measurement module is composed of the power-measuring integrated circuit (IC) 71M6541D, which is a Teridian 4th generation single-phase metering system on a chip (SoC), with an error margin of 0.1% that meets all ANSI and IEC electricity metering standards. This IC is an integrated power-measurement device that combines a 22-bit second-order delta-sigma analog-to-digital converter (ADC), four analog inputs, digital temperature compensation, a precision voltage reference, an independent 32-bit computation engine (CE), and a serial interface (SPI) on a single chip. Additional features include AC and DC calibration and phase compensation. Designed for residential single-phase or industrial three-phase power-meter applications, the IC accurately measures the instantaneous current and voltage while calculating the root-mean-square voltage *U*_rms_, root-mean-square current *I*_rms_, reactive power *Q*, active power *P*, apparent power *S*, power factor PF, total voltage harmonic distortion *U*_THD_, total current harmonic distortion *I*_THD_, and so on [27]. The circuit of this module is shown in Figure 6.

The flow chart for calculating the RMS voltage and current can be divided into two parts: AD conversion and digital signal processing [25].

*Part 1 (Analog-to-Digital Conversion)*. An ADC is used to convert an analog signal into a digital signal. The measuring IC has a 22-bit second-order sigma-delta ADC, which is used to convert the voltage signal and current signal. The ADC output is decimated by the finite impulse response (FIR) filter and is stored in CE random access memory (RAM), where it can be accessed and processed by the CE. The maximum signal input into the measuring IC is ±0.25 V. Therefore, a high-resistance voltage divider with a properly designed resistance, which can convert AC 250 V into ±0.25 V, is used. In addition, the current sampling can use a current transformer to measure the current signal with proper resistance and can convert AC 30 A into ±0.25 V. The converted voltage and current are input into the measuring IC to proceed to the next calculation, as shown in Figure 6.

*Part 2 (Digital Signal Processing)*. Various power parameters can be computed in real time, including the root-mean-square voltage *U*_rms_, root-mean-square current *I*_rms_, reactive power *Q*, active power *P*, apparent power *S*, power factor PF, total voltage harmonic distortion *U*_THD_, and total current harmonic distortion *I*_THD_. The formulations adopted for computing the power parameters are described below [28].

Although the fast Fourier transform (FFT) is efficient, it has a strict requirement for the collected data; namely, the number of collected data points must be exactly a power of 2 (2^*n*^) [29]. If we use the FFT to process data, the reducing spectrum leakage problem or the hurdle effect is likely to arise, which does not suit our goals. Thus, in this system, we use the discrete Fourier transform (DFT) algorithm, which requires only 100 data samples/points during a period and has an acceptable time range, instead of using the FFT to process the collected data [30].

Assume that a voltage or current signal *x*(*t*) having a sampled sequence *x*(*n*) is sampled at a regular time interval *T*, that is, {*x*(0), *x*(*T*),…, *x*((*N* − 1)*T*)}. The DFT of *x*(*n*) is *X*(*k*), defined as the sequence of complex values {*X*(0), *X*(*w*_0_),…, *X*((*N* − 1)*w*_0_)} in the frequency domain, where *w*_0_ is the fundamental frequency given by *w*_0_ = 2*π*/*NT*. According to the decimation-in-time FFT algorithm [31], the DFT values *X*(*k*) at frequency *kw*_0_ are computed as follows:

(1) $$\begin{array}{c} X\left(k\right)=\overset{N-1}{\underset{n=0}{\sum }}x\left(n\right)W_{N}^{nk},\;k=0,1,\ldots ,N-1, \end{array}$$

where *W*_*N*_^*nk*^ = *e* ^−*j*(2*π*/*N*)*kn*^ is the twiddle factor.

Given the DFT values *U*(*k*) and *I*(*k*), the root-mean-square values *U*_rms_ and *I*_rms_ of the sampled voltage and the current values *u*(*n*) and *i*(*n*) can be computed as follows:

(2) Urms2=2N2{∑k=0N/2−1((Re[U(k)])2+(Im⁡[U(k)])2)},Irms2=2N2{∑k=0N/2−1((Re[I(k)])2+(Im⁡[I(k)])2)},

where Re[*·*] and Im[*·*] represent the real and imaginary parts, respectively.

The reactive power *Q* and the active power *P* can be computed as follows:

(3) P=2N2{∑k=0N/2−1((Re[U(k)]Re[I(k)])  +(Im⁡[U(k)]Im⁡[I(k)]))},Q=2N2{∑k=0N/2−1((Re[I(k)]Im⁡[U(k)])  −(Re[U(k)]Im⁡[I(k)]))}.

The apparent power *S* and the power factor PF are calculated as follows:

(4) $$\begin{array}{c} S=U_{\text{rms}}I_{\text{rms}},\\ \text{PF}=\frac{p}{s}. \end{array}$$

Finally, the total voltage harmonic distortion *U*_THD_ and the total current harmonic distortion *I*_THD_ are computed as follows:

(5) $$\begin{array}{c} U_{\text{THD}}=\frac{\sqrt{\sum_{k=2}^{N/2}U^{2}\left(k\right)}}{U\left(1\right)}\times 100\%,\\ I_{\text{THD}}=\frac{\sqrt{\sum_{k=2}^{N/2}I^{2}\left(k\right)}}{I\left(1\right)}\times 100\%. \end{array}$$

#### 3.3.3. Control Module

The control module includes a relay and its driving circuit, as shown in Figure 6. This module mainly receives control instructions from the ZigBee module to acquire the status of the relay and to then control the output power of the outlet. The controller signal from the ZigBee module is amplified by the transistor and is then transmitted to the drive relay. The freewheeling diodes set on both sides of the relay are used to provide a release method for the diode to generate a reversed voltage, instantly changing the relay from ON to OFF and preventing damage to the transistor [25].

**Figure 7:**
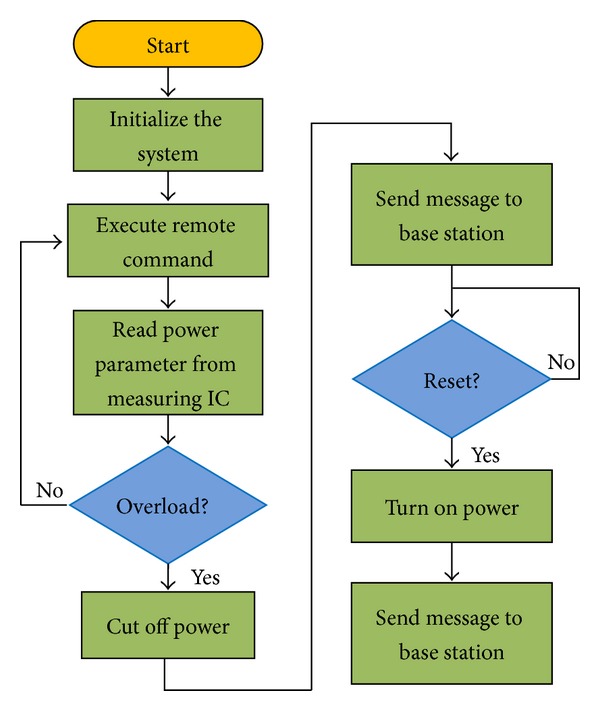
Flow chart of the sensor node.

Figure 7 shows a software flow chart of the sensor node.

**Figure 8:**
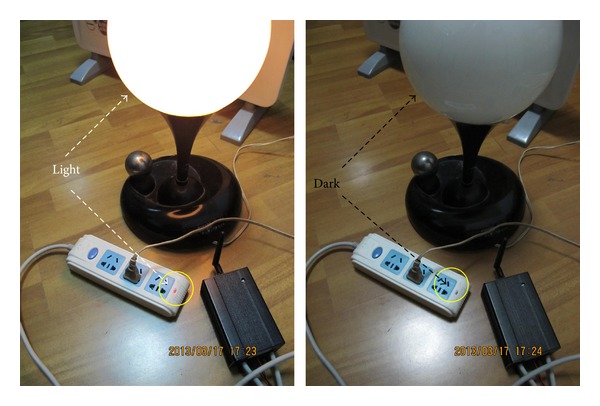
Control module in action.

Figure 8 displays the function of the control module.

#### 3.3.4. ZigBee Module

The ZigBee module is composed of mote processor/radio platforms (XM2110), which use the Atmel RF230, IEEE 802.15.4 compliant, ZigBee-ready radio frequency transceiver integrated with an Atmega1281 MCU. These enhancements provide up to three times the radio range and twice the program memory of previous-generation MICA motes [32]. A block diagram of XM2110 is shown in Figure 2. In the sensor node, XM2110 connects with the sensor board via a 51-pin expansion connector, whose structure is shown in Figure 6.

ZigBee is a wireless network protocol and an adapted IEEE 802.15.4 standard owned by ZigBee Alliance, which defines the media layer and the objective layer. ZigBee exhibits low transmission speed at low cost and low energy consumption, with high security, and supports a large number of web node operations. Therefore, ZigBee is very suitable for use in building monitoring and controlling systems.

In the ZigBee module, the effective transmission distance between nodes is determined by the transmission energy designed for the module. At present, the transmission distance of the commercial module can reach approximately 100 m under the barrier-free condition. Although the partition blocks of buildings may reduce the communication distance, the use of ZigBee can support the network structure with a tree or mesh, and setting certain nodes in the network to the router function can effectively overcome the issues of transmission in the same horizontal floor and at different vertical floors over a long distance. Conceptually, ZigBee communication can be applied to buildings without restrictions on the transmission distance [25, 33].

To help resolve the noise interference issue, ZigBee uses the direct sequence spread spectrum (DSSS) to reduce the environmental interference and uses a carrier sense multiple access with collision avoidance (CSMA/CA) channel access mechanism, dynamic frequency selection, and transmission power control to avoid channel collisions [4, 34, 35].

## 4. Experimental Results

### 4.1. Accuracy Verification

**Figure 9:**
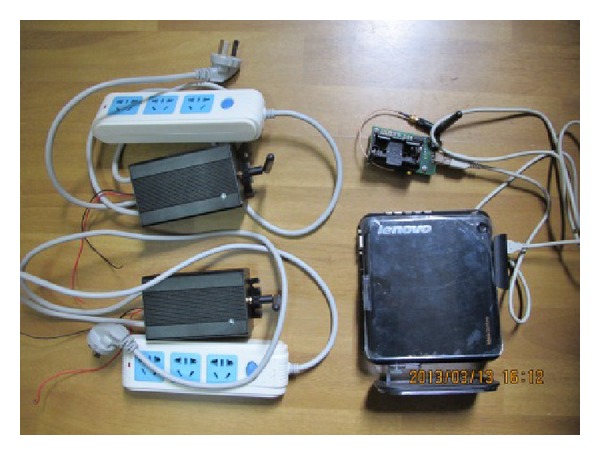
Prototype system.

**Figure 10:**
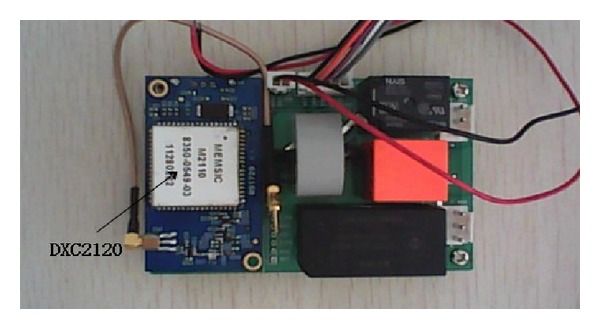
Circuits of the sensor node.

**Figure 11:**
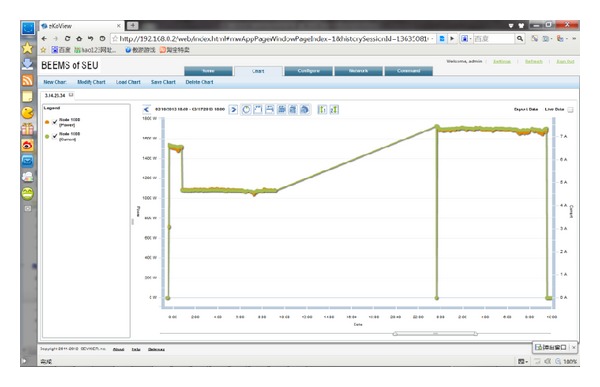
GUI showing the local and remote monitoring and control screen.

To verify the accuracy and implementation of the ZBEMCS, a practical demonstration system was produced; the methods, design procedures, and practical work are shown in Figure 9. This demonstration system is equipped with one base station that manages two branch circuits, each of which includes one sensor node. Each smart node is composed of a ZigBee module, a MCU, a power-measuring IC, voltage and current measure circuits, and relays. The physical system is shown in Figure 10. In this system, VB.NET is used to program the user interface of the remote monitoring and control center, which would communicate with the base station through Internet. The operating screen is shown in Figure 11.

**Figure 12:**
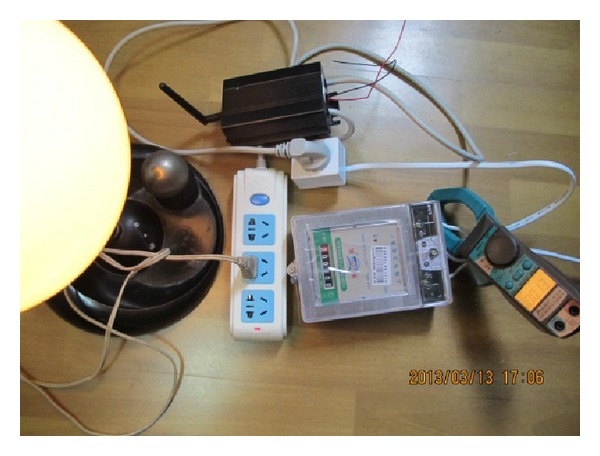
ZBEMCS, standard wattmeter, and clamp meter.

**Figure 13:**
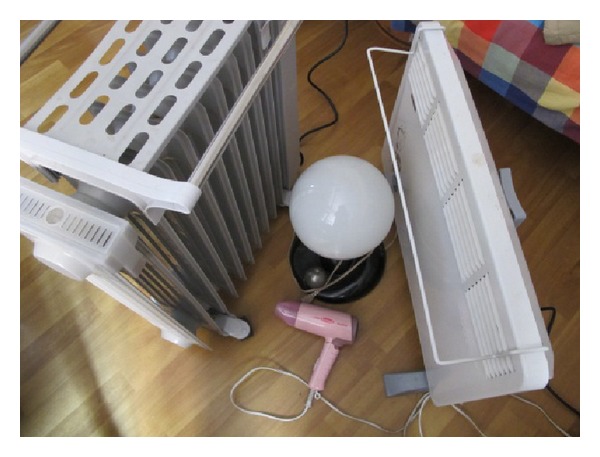
The tested appliances included a lamp, a hair dryer, and two electric heaters.

First, we use the ZBEMCS and a standard clamp meter to measure the current and voltage usage of a lamp, a hair dryer, and an electric heater simultaneously, as shown in Figures 12 and 13.  Table 2 indicates the experimental results. From Table 2, we can see that the average deviation of the measured current between the ZBEMCS and the standard clamp meter is 0.051 A, and the average deviation of the voltage is 2.0 V; the corresponding standard deviation of the current is 0.017 A, and that of the voltage is 0.545 V.

Next, we use the ZBEMCS and a standard wattmeter to measure the energy consumption of two electric heaters. The experimental results are shown in Table 3. From this table, we find that although the measurement values of the ZBEMCS and the standard wattmeter are both larger than those of the nominal power, the average deviation and standard deviation between them are negligible. The average deviation of branch 1 is 0.058 kW*·*h, that of branch 2 is 0.060 kW*·*h, and that of the trunk is 0.118 kW*·*h; the standard deviation of branch 1 is 0.035 kW*·*h, that of branch 2 is 0.035 kW*·*h, and that of the trunk is 0.071 kW*·*h.

Therefore, the measurement accuracy of the ZBEMCS is dependable, and we can use the ZBEMCS to monitor the parameters of building electric devices.

### 4.2. Case Study

**Figure 14:**
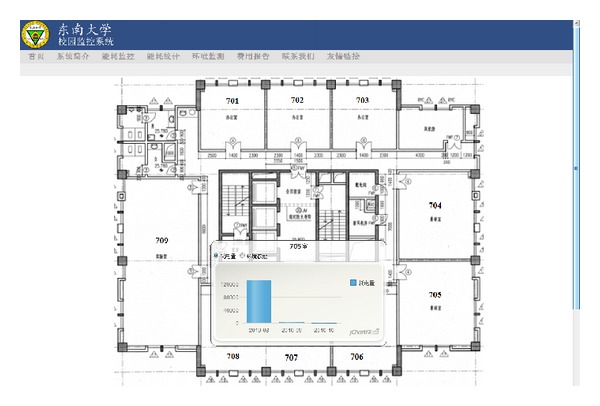
Floor plan of the seventh floor of the RRSAB.

**Figure 15:**
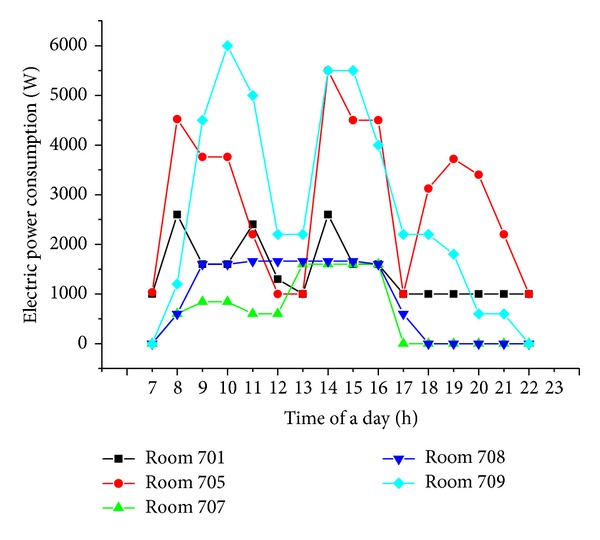
Electric power consumption of the testing rooms.

The Run Run Shaw Architectural building (RRSAB) is an office building located at Southeast University, Nanjing, China. We use the ZBEMCS to monitor the power consumed by the electric devices in the rooms 701, 705, 707, 708, and 709 of the 7th floor of the RRSAB.  Figure 14 shows the floor plan of the seventh floor of the RRSAB.  Table 4 lists the facilities in the testing rooms.  Figure 15 shows electric power consumptions of the testing rooms.

From Figure 15, the power consumptions for every hour of the facilities in the testing rooms are easily available.

## 5. Application of ZBEMCS

**Figure 16:**
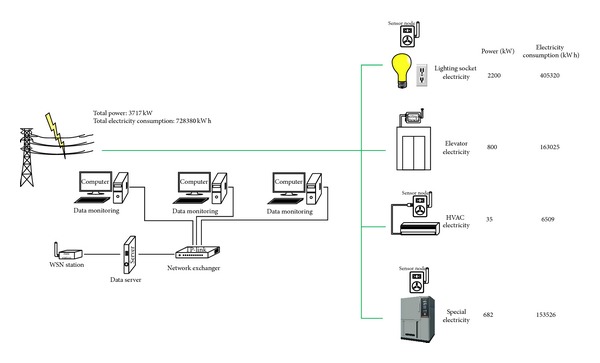
Subentry metering of building energy by ZBEMCS.

ZBEMCS provides both local/remote power parameter measurement and power on/off switching for electric appliances. Therefore, this system can be used for energy consumption monitoring, long-term energy conservation planning, and the development of automated energy conservation for building applications. One typical application of ZBEMCS is subentry metering of building energy. For example, for most campus buildings whose energy consumed is mainly power in hot and humid climate, ZBEMCS collects the data according to the electricity system, which can be divided into the following four separate items, as shown in Figure 16:

1. lighting socket electricity, which mainly includes the lighting and power sockets (indoor lighting electricity, air terminal socket electricity, and regular socket electricity), the corridor and emergency light electricity, and the outdoor landscape lighting;
2. HVAC (heating, ventilation, and air conditioning) electricity, which mainly includes the electricity of the heating and cooling source equipment (refrigerating electricity, fan electricity of the cooling tower, and electricity of the electric boiler), air terminal socket electricity, and electricity of the transportation equipment (chilled water pump, cooling water pump, and hot water circulation pump electricity);
3. power electricity, which mainly includes the electricity for the elevator, water pump, fan, and special electricity (where special electricity refers to the special power consumption that does not belong to the normal function of the electrical equipment). The special characteristic of special electricity is a high energy density; it uses more power than the major electricity facilities and equipment. According to the campus building characteristics, special electric facilities generally include laboratories, clean rooms, information centers, dining rooms, laundry rooms, swimming pools, and other special facilities;
4. special electricity for large special equipment for scientific research or other auxiliary equipment.

**Figure 17:**
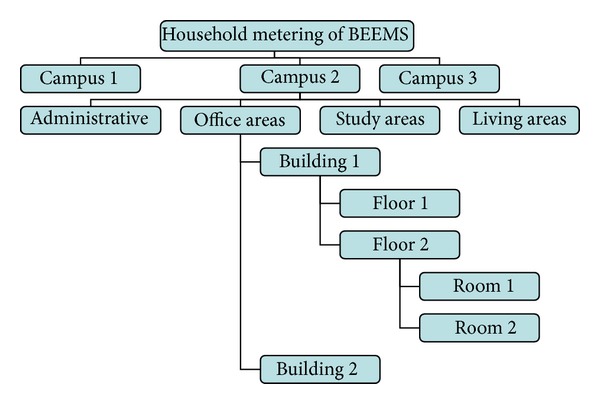
Household metering method of the ZBEMCS according to the campus main function.

Another typical application of ZBEMCS is household metering of building energy. For example, one household metering method is implemented according to the campus's main function zone, as shown in Figure 17. It considers the campus as an energy management subcenter and divides every campus building into four components: the administrative area, office area, study area, and living area. Then, it finds the sum of the energy consumption of each building. We perform the statistics step by step from the room to building, namely, in the order of the classroom, floors, building, and energy management center.

**Figure 18:**
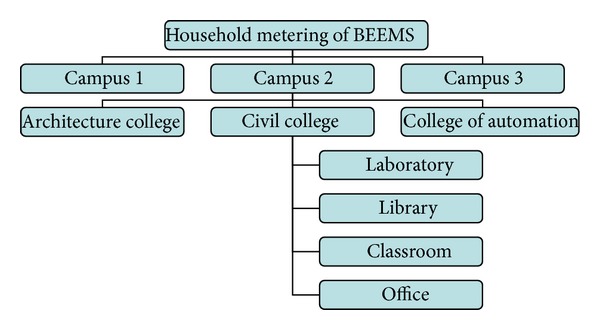
Household metering method of the ZBEMCS according to each college.

The other household metering method is implemented according to each college or department, as shown in Figure 18, which is campus-college-function-energy consumption monitoring. The specific metering method is similar to the first method. It is applicable to the campus that needs separate metering for every college. In particular, this method divides the campus into different small campuses and builds a subcenter of the building energy consumption monitoring system so that each small campus can independently monitor its energy consumption. Each small campus can build its own college energy consumption monitoring system. In addition, each campus can also be divided into an administrative area, a teaching area, a library, a living area, an office area, and other different areas. Then, a subset partition system of the energy consumption monitoring system is established. To improve the management convenience, the office area and laboratory can be allocated to the college management and monitoring system and conducted by each college. We also perform the statistics step by step from rooms to buildings using the same statistical method as in the first method.

## 6. Conclusions

In this paper, the authors propose a cost-effective ZBEMCS, which consists of a gateway, a base station, and sensors. Specifically, a new hardware platform for power sensor nodes is developed to perform both local/remote power parameter measurement and power on/off switching for electric appliances. The experimental results demonstrate that the ZBEMCS can easily monitor energy usage with a high level of accuracy. Two typical applications of ZBEMCS such as subentry metering and household metering of building energy are presented. The former includes lighting socket electricity, HVAC electricity, power electricity, and special electricity. The latter includes household metering according to the campus's main function zone and each college or department. Therefore, this system can be used for energy consumption monitoring, long-term energy conservation planning, and the development of automated energy conservation for building applications.

## Figures and Tables

**Table 1:** Key characteristics of common wireless standards.

	Data rate	Range	Network topology	Operating frequency	Complexity	Power consumption	Security	Other information	Typical applications
ZiBee	20, 40, and 250 kb/s	10–100 m	Ad hoc, peer-to-peer, star, or mesh	868 MHz (Europe) 900–928 MHz (NA), 2.4 GHz (worldwide)	Low	Very low	128-AES plus application layer security	Devices can join an existing network in under 30 ms	Industrial control and monitoring, sensor networks, building automation, home control and automation, toys, and games

Wi-Fi	11 and 54 Mb/s	50–100 m	Point-to-hub	2.4 and 5 GHz	High	High		Device connection requires 3–5 s	Wireless local area network (LAN) connectivity, broadband Internet access

Bluetooth	1 Mb/s	10 m	Ad hoc, very small networks	2.4 GHz	High	Medium	64- and 128-b encryption	Device connection requires up to 10 s	Wireless connectivity between devices, such as phones, personal digital assistants (PDAs), laptops, and headsets

BLE	1 Mb/s	>100 m	Ad hoc, very small networks	2.4 GHz	High	Low	128-bit AES with counter Mode CBC-MAC	6 ms latency from a non-connected state	Mobile phones, gaming, watches, healthcare, and home electronics

UWB	100–500 Mb/s	<10 m	Point-to-point	3.1–10.6 GHz	Medium	Low			Streaming video, home entertainment applications

Wireless USB	62.5 kb/s	10 m	Point-to-point	2.4 GHz	Low	Low			Peripheral PC connections

IR Wire-less	20–40 kb/s 115 kb/s 4 and 16 Mb/s	<10 m (line of sight)	Point-to-point	800–900 nm	Low	Low			Remote controls, PCs, PDAs, phones, and laptop links

**Table 2:** Current and voltage data comparison between the ZBEMCS and a standard clamp meter.

	A: ZBEMCS		B: standard clamp meter		A−B	
	Current (A)	Voltage (V)	Current (A)	Voltage (V)	Current (A)	Voltage (V)
Lamp	0.27	235.2	0.25	238	0.02	−2.8
	0.26	235.5	0.24	238.2	0.02	−2.7
	0.25	235.5	0.22	237.2	0.03	−1.7
	0.24	235.3	0.19	236.4	0.05	−1.1
	0.23	235	0.18	236.5	0.05	−1.5
	0.22	235	0.16	236.5	0.06	−1.5
	0.21	233.9	0.15	236.6	0.06	−2.7
	0.2	234	0.14	236.7	0.06	−2.7

Hair dryer	0.77	235.4	0.72	237.4	0.05	−2.0
	3.53	233.8	3.46	236.1	0.07	−2.3
	4.49	233.8	4.42	235.5	0.07	−1.7

Electric heater	2.82	234.5	2.75	236.3	0.07	−1.8
	4.71	233.2	4.65	235	0.06	−1.8
	7.5	231.7	7.45	233.4	0.05	−1.7

Average deviation					0.051	−2.0

Standard deviation					0.017	0.545

**Table 3:** Power consumption data for two electric heaters (kWh).

Time (h)		1.00	8.17	9.35		

C: ZBEMCS	Branch 1	0.609	8.838	15.779		
	Branch 2	1.508	18.633	7.278		
	Trunk	2.117	27.471	23.057		

D: standard wattmeter	Branch 1	0.7	8.9	15.8		
	Branch 2	1.6	18.7	7.3		
	Trunk	2.3	27.6	23.1		

Power on nameplate (kW)	Branch 1	0.6	1.0	1.6		
	Branch 2	1.5	2.2	0.7		
	Trunk	2.1	3.2	2.3		

E: nominal power	Branch 1	0.6	8.170	14.960		
	Branch 2	1.5	17.974	6.545		
	Trunk	2.1	26.144	21.505	Average deviation	Standard deviation

C−D	Branch 1	−0.091	−0.062	−0.021	−0.058	0.035
	Branch 2	−0.092	−0.067	−0.022	−0.060	0.035
	Trunk	−0.183	−0.129	−0.043	−0.118	0.071

C−E	Branch 1	0.009	0.668	0.819	0.499	0.431
	Branch 2	0.008	0.659	0.733	0.467	0.399
	Trunk	0.017	1.327	1.552	0.965	0.829

D−E	Branch 1	0.100	0.730	0.840	0.557	0.399
	Branch 2	0.100	0.726	0.755	0.527	0.370
	Trunk	0.200	1.456	1.595	1.084	0.768

**Table 4:** The facilities in the testing rooms.

Rooms	Facilities
701 Office	2 computers, 1 air condition, 1 electric kettle, 1 refrigerator, 1 fountain, 1 water dispenser, and 1 microwave oven

705 Teaching room	11 computers, 4 fans, 1 refrigerator, 1 air condition, and 1 electric kettle

707 Reading room	1 computer, 1 air condition, 4 fans, and 1 water dispenser

708 Library	2 computers, 1 air condition, and 1 fan

709 Lab	4 computers, 1 air condition, 1 electric kettle, 1 water dispenser, and 3 robots
