# Corrigendum to “Development and Application of a ZigBee-Based Building Energy Monitoring and Control System”

**DOI:** 10.1155/2017/3178673

**Published:** 2017-07-12

**Authors:** Changhai Peng, Kun Qian

**Affiliations:** ^1^School of Architecture, Southeast University, Nanjing 210096, China; ^2^Key Laboratory of Urban and Architectural Heritage Conservation (Southeast University), Ministry of Education, Nanjing 210096, China; ^3^IIUSE, Southeast University, Nanjing 210096, China; ^4^School of Automation, Southeast University, Nanjing 210096, China

In the article titled “Development and Application of a ZigBee-Based Building Energy Monitoring and Control System” [1], the authors based their article on two of their previous publications [2, 3] that are not cited. The four numbered items in the section titled “5. Application of ZBEMCS” in our article [1] are reproduced from the section titled “3.1. Classification and Subentry Metering of the BEEMS” in article [2].

Additionally, there are three figures in article [1] that are taken from the previous publications [2, 3], where Figures 17 and 18 correspond to Figures 5 and 6 in article [2], respectively, and Figure 14 is the same as Figure 5 in article [3].
